# Ultrasound-Assisted Enantioselective Esterification of Ibuprofen Catalyzed by a Flower-Like Nanobioreactor

**DOI:** 10.3390/molecules21050565

**Published:** 2016-04-28

**Authors:** Baiyi An, Hailin Fan, Zhuofu Wu, Lu Zheng, Lei Wang, Zhi Wang, Guang Chen

**Affiliations:** 1 College of Horticulture, Jilin Agricultural University, Changchun 130118, China; swabyswaby@163.com; 2 College of Resources and Environment, Jilin Agricultural University, Changchun 130118, China; fanhl@jlau.edu.cn; 3 College of Life Science, Jilin Agricultural University, Changchun 130118, China; wuzhuofu@163.com; 4 Key Laboratory of Molecular Enzymology and Engineering of Ministry of Education, School of Life Sciences, Jilin University, Changchun 130012, China; zhengl14@mails.jlu.edu.cn (L.Z.); w_lei@jlu.edu.cn (L.W.)

**Keywords:** ibuprofen, APE1547, resolution, ultrasound, nanobioreactor

## Abstract

A flower-like nanobioreactor was prepared for resolution of ibuprofen in organic solvents. Ultrasound irradiation has been used to improve the enzyme performance of APE1547 (a thermophilic esterase from the *archaeon Aeropyrum pernix* K1) in the enantioselective esterification. Under optimum reaction conditions (ultrasound power, 225 W; temperature, 45 °C; water activity, 0.21), the immobilized APE1547 showed an excellent catalytic performance (enzyme activity, 13.26 μmol/h/mg; *E* value, 147.1). After ten repeated reaction batches, the nanobioreactor retained almost 100% of its initial enzyme activity and enantioselectivity. These results indicated that the combination of the immobilization method and ultrasound irradiation can enhance the enzyme performance dramatically.

## 1. Introduction

Ibuprofen is a nonsteroidal drug and has an asymmetric carbon in its second position. It’s known that its bioactivity mainly resides in its *S*-enantiomer [1]. Therefore, the synthesis of (*S*)- ibuprofen is strongly recommended. It is well known that chemical synthesis is the classical method for preparing the chiral compounds, but it is usually conducted using asymmetric synthesis catalyzed by expensive catalysts, which are inefficient, laborious and toxic. Compared with the chemical synthesis, many biocatalytic processes have been developed and turned out to be more attractive for obtaining the chiral compounds with high enantiopurity [2,3,4]. Among the biocatalytic processes, enzyme-catalyzed kinetic resolution is one of the most efficient methods [5]. In our previous study [6], we successfully used a thermophilic esterase (APE1547) from the *archaeon Aeropyrum pernix* K1 to carry out the enantioselective esterification of ibuprofen. We also found that ultrasound can be adopted to improve the biocatalytic properties of APE1547 in the resolution of ibuprofen [7]. However, the enzyme performance of thermophilic esterase (APE1547) is still not satisfacctory and the used enzyme could not be recycled.

It’s known that immobilization is a powerful tool to avoid the enzyme aggregation in organic solvents and can enhance the reusability. Furthermore, immobilization may also improve other enzyme properties, such as stability, selectivity or specificity [8,9,10,11,12,13,14]. However, many immobilized enzymes can not be used efficiently under high-speed agitation or under ultrasound [15,16]. For example, some immobilized enzymes are prepared by physical adsorption, but, high-speed agitation or ultrasound can destroy the weak interactions between the enzyme and the immobilized support, which will induce enzyme loss during the reaction cycles and inactivate the enzyme [14]. Some enzymes are immobilized on fragile supports with poor operational stability [17]. Both high-speed agitation and ultrasound can cause the collapse of the immobilized support and affect the enzyme performance of the immobilized enzyme. In 2014, we prepared a flower-like nanobioreactor which was formed *via* the coordination between Cu^2+^ and the nitrogen atoms of the amide groups in the enzymes [18]. The simple and efficient preparation procedure does not require expensive facilities and the preparation cost is low. Furthermore, our unpublished study has demonstrated that this kind of immobilized enzyme is very stable under many extreme environments (low or high pH, high temperature, organic reaction media, high-speed agitation or ultrasound, *et al.*). These promising advantages make this immobilization method more attractive for further study.

**Scheme 1:**
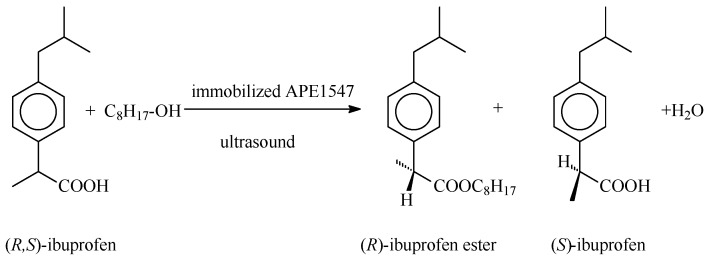
Schematic diagram of the resolution of ibuprofen.

In this study, we combined this immobilization method and ultrasound irradiation to improve the enzyme performance in enantioselective esterification of ibuprofen (Scheme 1). Furthermore, the stability and reusability of the APE1547-incorporated nanobioreactor under ultrasound irradiation had also been studied.

## 2. Results and Discussion

### 2.1. The Morphologies of the Immobilized Samples

**Figure 1:**
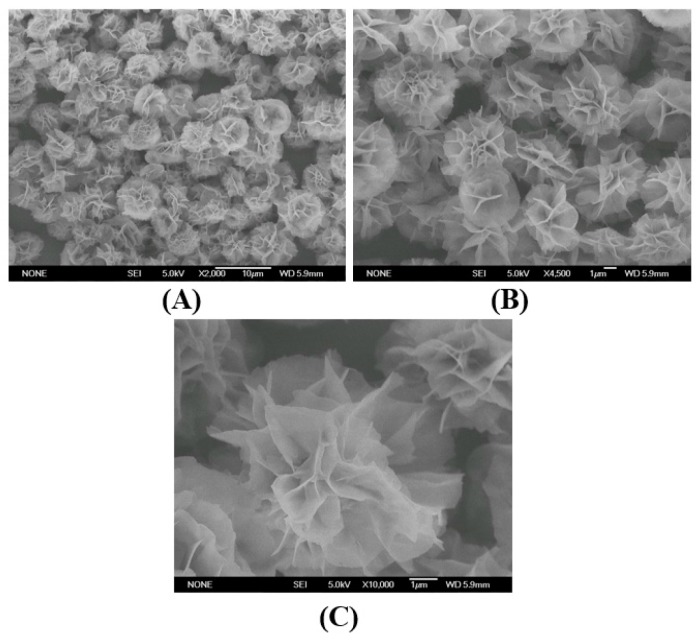
The morphologies of the immobilized samples. (**A**–**C**): SEM images of the immobilized samples with different enlargement factors.

A large number of immobilized APE1547 have been successfully prepared (Figure 1A,B). The shape of the immobilized enzyme looked like a natural flower, with dozens of separate petals with high surface (Figure 1C). Its diameter was about 4–5 μm. The excellent hierarchical structure with high surface to volume ratios is likely to improve the enzyme performance of the immobilized enzyme.

### 2.2. FTIR

**Figure 2:**
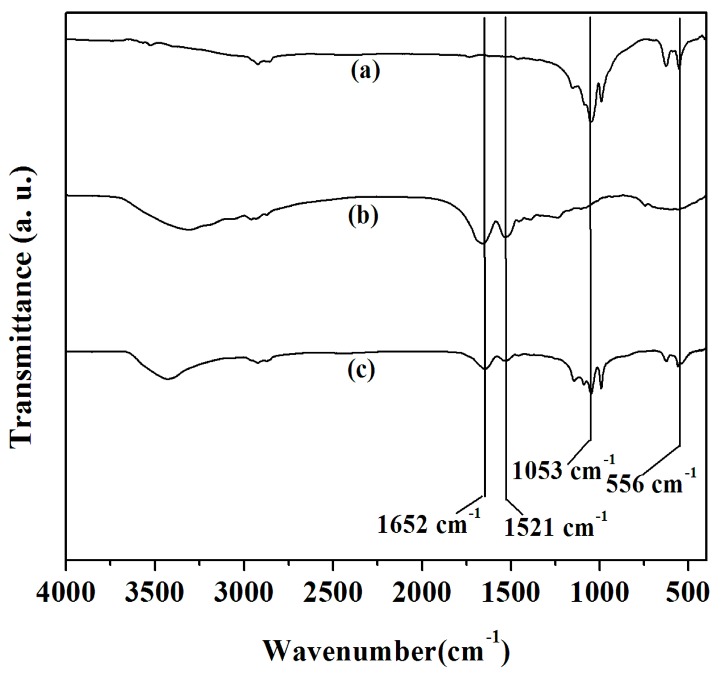
The FTIR spectrum of Cu_3_(PO_4_)_2_ matrices (curve a), APE1547 (curve b) and the immobilized APE1547 (curve c).

The FTIR spectra of the immobilized samples were recorded and the results are shown in Figure 2. Several characteristic peaks could be observed in curve c. Among of them, the peaks at 1053 cm^−1^ and 556 cm^−1^ are assigned to the vibrations of PO_4_^3−^ [19]. The peaks at 1652 cm^−1^ and 1521 cm^−1^ are ascribed to the vibrations of the amide I and II bands of the enzyme [20]. These results successfully verified the presence of enzyme in the immobilized samples.

### 2.3. Effect of Ultrasound Power

**Figure 3:**
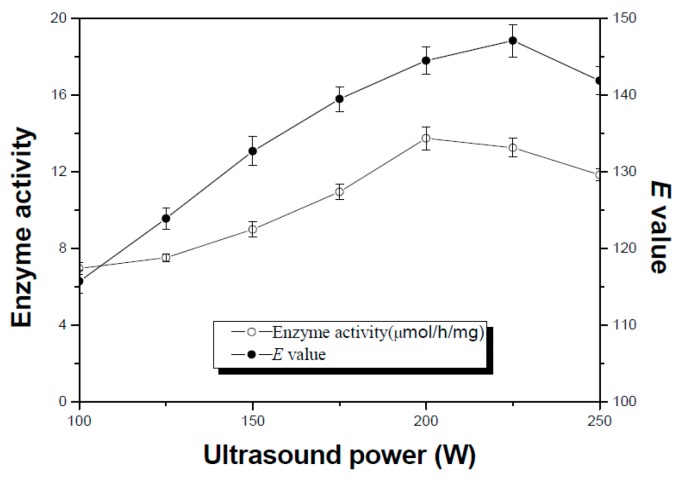
Effect of ultrasound power on the enzymatic esterification of ibuprofen. Reaction conditions: the reactions were carried out in *n*-heptane (10 mL, *a*_w_ 0.21) with racemic ibuprofen (0.2 mmol), 1-octanol (0.2 mmol) and the immobilized APE1547 powder (160 mg) at 45 °C.

The effect of ultrasound power on the enantioselective esterification was examined and the results are shown in Figure 3. The highest enantioselectivity was obtained at an ultrasound power of 225 W and the maximum enzyme activity could be observed at 200 W. The enhancement of enzyme activity obtained at suitable ultrasound power (225 W) was probably attributed to the better mixing of the reactants [21]. Furthermore, the improvement of the mass transfer processes in the nanobioreactor can also increase the enzyme activity considering the complexity and heterogeneity of the immobilized enzyme. However, the mechanism for the ultrasound-induced alteration in the enantioselectivity is still unclear. Our previous study has demonstrated that ultrasound can induce a slight change of the secondary structure of APE1547 and then influence its enantioselectivity [7]. Further research is needed to clarify the mechanism and will be published in due course.

### 2.4. Effect of Temperature

**Figure 4:**
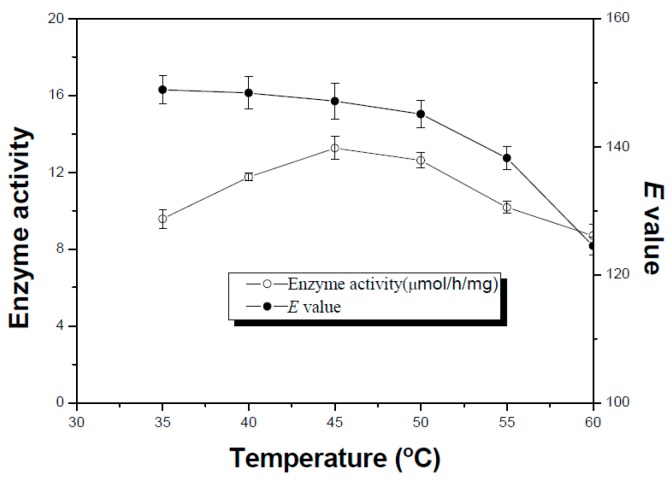
Effects of temperature on the enzymatic esterification of ibuprofen. Reaction conditions: the reactions were carried out in *n*-heptane (10 mL, *a*_w_ 0.21) with racemic ibuprofen (0.2 mmol), 1-octanol (0.2 mmol) and the immobilized APE1547 powder (160 mg) at different temperature. The ultrasound power was 225 W.

The effects of ultrasound temperature on the enzyme performance have also been investigated in this study. The result in Figure 4 showed that higher enantioselectivity could be obtained at lower temperature. The possible explanation is that high temperature may destroy the conformation of the enzyme by heat-induced destruction of non-covalent interactions and decrease the enantioselectivity [22,23]. The enzyme activity exhibited a bell shaped curve with the changing ultrasound temperature, and the optimal temperature was 45 °C.

High reaction temperatures may elevate the collision probability between enzyme and substrate molecules to form enzyme-substrate complexes and then enhance the enzyme activity. The activity significantly decreased at higher temperatures, which might be due to the denaturation of the enzyme, especially under ultrasound irradiation [23]. Considering both the enzyme activity and enantioselectivity, 45 °C was selected as the optimal reaction temperature.

### 2.5. Effect of Water Activity

**Figure 5:**
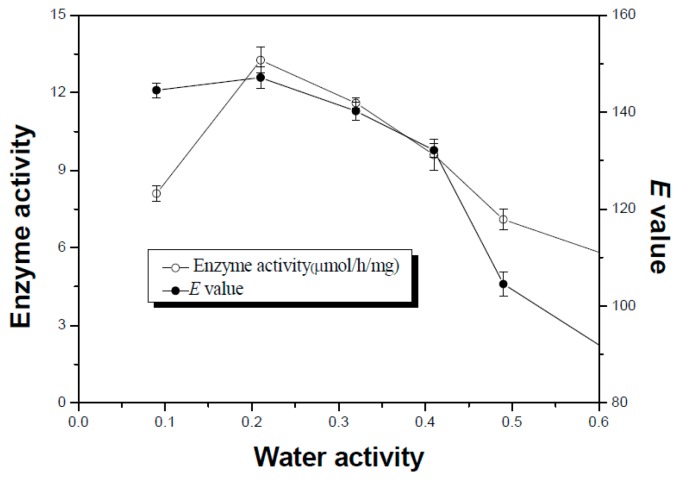
Effect of water activity on the enzymatic esterification of ibuprofen. Reaction conditions: the reactions were carried out in *n*-heptane (10 mL) with racemic ibuprofen (0.2 mmol), 1-octanol (0.2 mmol) and the immobilized APE1547 powder (160 mg) at 45 °C. The ultrasound power was 225 W.

Water may influence the performance of the enzymes in organic solvents [24]. The results in Figure 5 showed that the enzyme activity increased as water activity increased from 0.09 to 0.21, then decreased at higher water activity. The maximum enantioselectivity (*E* value) could also be observed at *a*_w_ = 0.21. Water activity may influence the reaction rates of the two isomers and then induce the alteration of enantioselectivity of APE1547 [24]. Since the *E* value (147.1) was found to be highest at *a*_w_ =0.21 while maintaining the highest enzyme activity (13.26 μmol/h/mg), the water activity of 0.21 was selected for further study.

### 2.6. Reusability and Stability 

For checking the reusability, the immobilized enzyme was used in ten continuous batches for the enzymatic esterification of ibuprofen under the optimal reaction conditions (ultrasound power, 225 W; temperature, 45 °C; water activity, 0.21). It could be found from Table 1 that the loss of the enzyme activity and enantioselectivity of the immobilized enzyme was almost negligible even after ten reaction cycles.

Furthermore, no change of the morphologies could be observed for the recycled immobilized nanobioreactor (Supplementary Figure S2). All these phenomena indicated that the prepared nanobioreactor was very stable under low power ultrasound conditions and exhibited an excellent reusability.

## 3. Experimental

### 3.1. Materials

(*S*)-(−)-1-(1-naphthyl) ethylamine ((*S*)-NEA), (*R*)-fenoprofen and racemic ibuprofen were purchased from Sigma-Aldrich-Fluka Chemical Co. (St. Louis, MO, USA). All other commercial reagents were from Shanghai Chemical Reagent Company, Shanghai, China. The (*S*)-NEA solution (0.02 M) was prepared in triethylamine-acetonitrile solution (10 mM). APE1547 was prepared in our lab [7,25].

### 3.2. Ultrasound Equipment

The ultrasound bath (KQ-250DE) was purchased from Kunshan Ultrasound Co. Ltd., Kunshan, China. Its maximum power is 250 W (scale, 40%–100%) and its temperature (±1 °C) is controlled by circulating water.

### 3.3. Preparation of the Nanobioreactor

The preparation of nanobioreactor was carried out according to our previous work [18]. The APE1547 solution (60 mL, 1 mg/mL) and CuSO_4_ solution (20 mL, 120 mmol/L) was mixed into a 3 L of PBS solution (50 mmol/L, pH 7.4). After incubation at 25 °C for three days, the blue precipitate at the bottom of the flash was collected by centrifugation (12,000 rpm for 20 min) and washed by deionized water for three times. The protein concentration in the supernatant was quantified by the Bradford protein assay [26] and the collected sample was about 960 mg. As a result, the immobilization yield of protein was determined. Since no protein in the pooled supernatant and washing solutions was detected, the immobilization yield of APE1547 was nearly 100% and the loading capacity of the nanoflowers was calculated to be 62.5 mg of protein per gram of nanoflowers.

### 3.4. Control and Measurement of Water Activity

All the reaction mixtures were previously dried under vacuum at 1 mm Hg for 12 h. Then, each reaction mixture with specific water activity (*a*_w_) was prepared through adding a specific volume of water. The resulting samples were pre-equilibrated at the desired temperature for 24 h in a sealed vial before being subjected to measurement of water activity (*a*_w_) with the Hygrolab Humidity Detector (Rotronic, Bassersdorf, Switzerland). 

### 3.5. Enzyme-Catalyzed Esterification of Ibuprofen 

Racemic ibuprofen (0.2 mmol), 1-octanol (0.2 mmol) and *n*-heptane (10 mL, *a*_w_ 0.21) were mixed in a 25-mL bottle. The bottle was incubated in the ultrasound (45 °C, 225 W). The immobilized APE1547 powder (160 mg) was added into the bottle to begin the enantioselective esterification. During the reaction, 50 μL of the reaction mixture was withdrawn from the bottle and derivatized with (*S*)-NEA (100 μL). After 3 min, the derivatization was stopped by adding 100 μL of ethanolamine solution (0.02 M in acetonitrile). Finally, the sample (about 20 μL) was withdrawn for HPLC analysis.

### 3.6. Characterization of the Prepared Nanobioreactor

The SEM of the samples was observed by a JSM-6700F electron microscope (JEOL, Tokyo, Japan) with an acceleration voltage of 30 kV. The FTIR spectra of the samples were surveyed using a 5700 FTIR spectrometer (Nicolet, Madison, WI, USA) with a resolution of 4 cm^−1^ through KBr method.

### 3.7. Recycling the Enzyme

After each batch (2.5 h), the reaction mixture was centrifuged at 10,000 rpm and 4 °C for 5 min. The precipitate (the immobilized APE1547) was washed at least three times with the reaction media and dried at room temperature.

### 3.8. High Performance Liquid Chromatography (HPLC) Analysis

An Agilent 1200 HPLC (Agilent, Santa Clara, CA, USA) equipped with a YMC C18 column (150 mm × 4.6 mm; Greenherbs Co. Ltd., Beijing, China) was used for HPLC detection (flow rate, 1.6 mL/min; 280 nm). The mixture of acetonitrile-water-acetic acid-triethylamine (volume ratio: 60:40:0.1:0.02; pH 5.0) was used as mobile phase. (*R*)-fenoprofen was used as an internal standard (retention time was 9.8 min). The retention time of the produced ibuprofen ester was 6.3 min. The retention time of the (*S*)-ibuprofen and (*R*)-ibuprofen was 15.1 and 16.8 min, respectively (Supplementary Figure S1). 

The degree of conversion (*C*) was calculated from the reduction of ibuprofen. One unit of the enzyme activity (μmol/h/mg) was defined as the amount (μmol) of ibuprofen ester produced per milligram of APE1547 containing in the immobilized sample per hour. According to our previous study [6], APE1547 favored the (*R*)-ibuprofen. The *ee* of the un-reacted (*S*)-ibuprofen and the enantiomeric ratio (*E* value) were calculated by the formula suggested by Chen *et al.* [27]:

(1) $$\begin{array}{c} enantiomeric\;excesses,ee(\%)=\frac{\left[S]-[R\right]}{\left[S]+[R\right]}\times 100\%\\ enantioselectivity.\;E=\frac{\ln[(1-C)(1-ee)]}{\ln[(1-C)(1+ee)]} \end{array}$$

In the above equation, [*S*] and [*R*] represent the concentration of the *S* and *R* isomer of ibuprofen, respectively.

## 4. Conclusions

In summary, we prepared a flower-like nanobioreactor for resolution of ibuprofen in organic solvents under ultrasound irradiation. After optimizing the reaction conditions, the nanobioreactor exhibited an excellent enzyme performance (enzyme activity, 13.26 μmol/h/mg; *E* value, 147.1). Compared with the native APE1547 in conventional reaction [6], the enzyme activity of the nanobioreactor under ultrasound was significantly enhanced about 61.25-fold and the enantioselectivity was enhanced about 3.86-fold. The most important finding is that the prepared nanobioreactor retained almost 100% of its initial enzyme activity and enantioselectivity, even after ten repeated reaction batches under ultrasound. These results demonstrated that ultrasound can enhance the enzyme performance dramatically and the prepared nanobioreactor can improve the reusability and stability of the used enzyme under ultrasound, which make this combination more attractive for further study. 

## Figures and Tables

**Table 1:** Reusability of the nanobioreactor under ultrasound.

Reaction Cycle	Relative Activity (%)	*E* value
1	100	147.1
2	99.6	150.9
3	99.5	148.4
4	98.7	146.3
5	99.2	151.4
6	99.6	147.2
7	98.8	148.5
8	98.7	151.3
9	99.1	146.5
10	99.5	147.7

## Supplementary Materials

Supplementary materials can be accessed at: http://www.mdpi.com/1420-3049/21/5/565/s1.
