# Supplementary material for: Ultrasound-Assisted Enantioselective Esterification of Ibuprofen Catalyzed by a Flower-Like Nanobioreactor
Source: Molecules. 2016 Apr 28;21(5):565. doi: 10.3390/molecules21050565 (PMC6273997; doi:10.3390/molecules21050565)
Supplement: Supplementary file 1 [file molecules-21-00565-s001.pdf]

Supplementary Materials: Ultrasound-Assisted Enantioselective Esterification of Ibuprofen Catalyzed by a Flower-Like Nanobioreactor

Baiyi An, Hailin Fan, Zhuofu Wu, Lu Zheng, Lei Wang, Zhi Wang and Guang Chen

High performance liquid chromatography (HPLC) Analysis

An Agilent 1200 HPLC equipped with a YMC C18 column (150 mm × 4.6 mm; Greenherbs Co. Ltd, Beijing, China) was used for HPLC detection (flow rate, 1.6 mL/min; 280 nm). The mixture of acetonitrile-water-acetic acid-triethylamine (volume ratio: 60:40:0.1:0.02; pH 5.0) was used as mobile phase. (*R*)-fenoprofen was used as an internal standard (retention time was 9.8 min). The retention time of the produced ibuprofen ester was 6.3 min. The retention time of the (*S*)-ibuprofen and (*R*)-ibuprofen was 15.1 and 16.8 min, respectively.

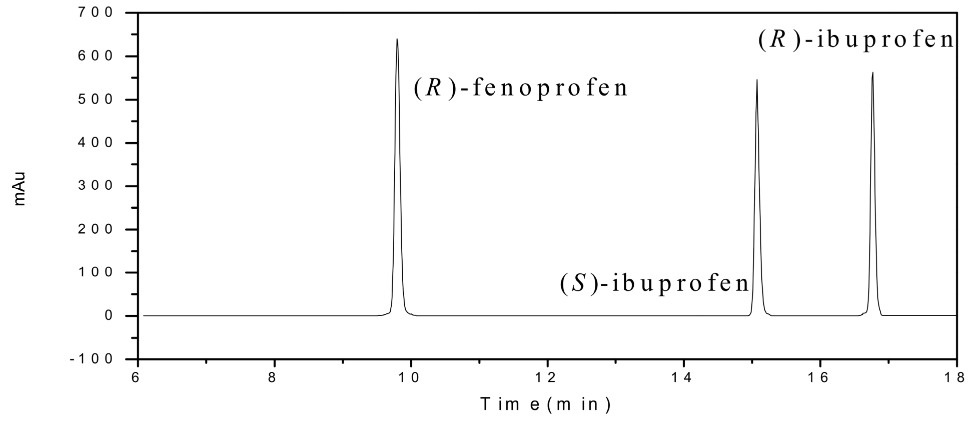

A

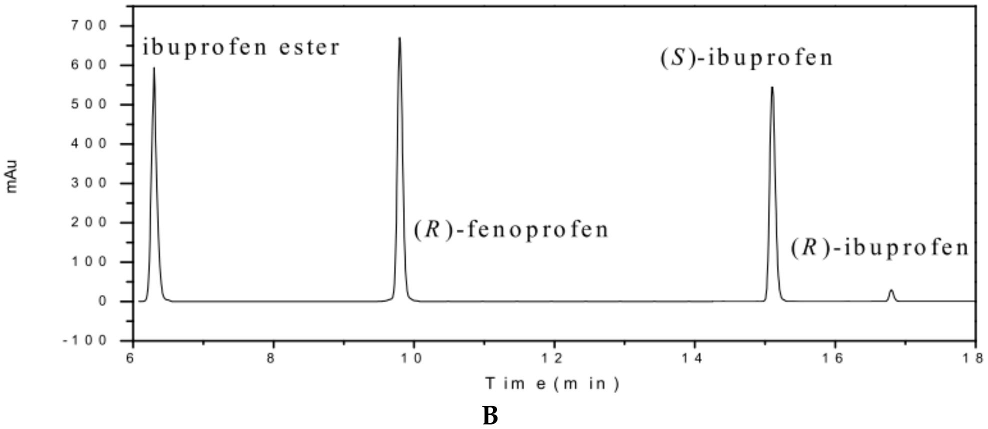

B

Figure S1. The chromatogram at the beginning of the reaction (before adding the enzyme) (**A**) and at the end of the bioconversion (**B**).

Electronic Supplementary Information

The SEM of the samples was observed by a JSM-6700F electron microscope (JEOL, Japan) with an acceleration voltage of 30 kV. The TEM of the samples was analyzed by using a FEI Tecnai G2 F20 s-twin D573 operated at 200 kV. Electronic Supplementary Information

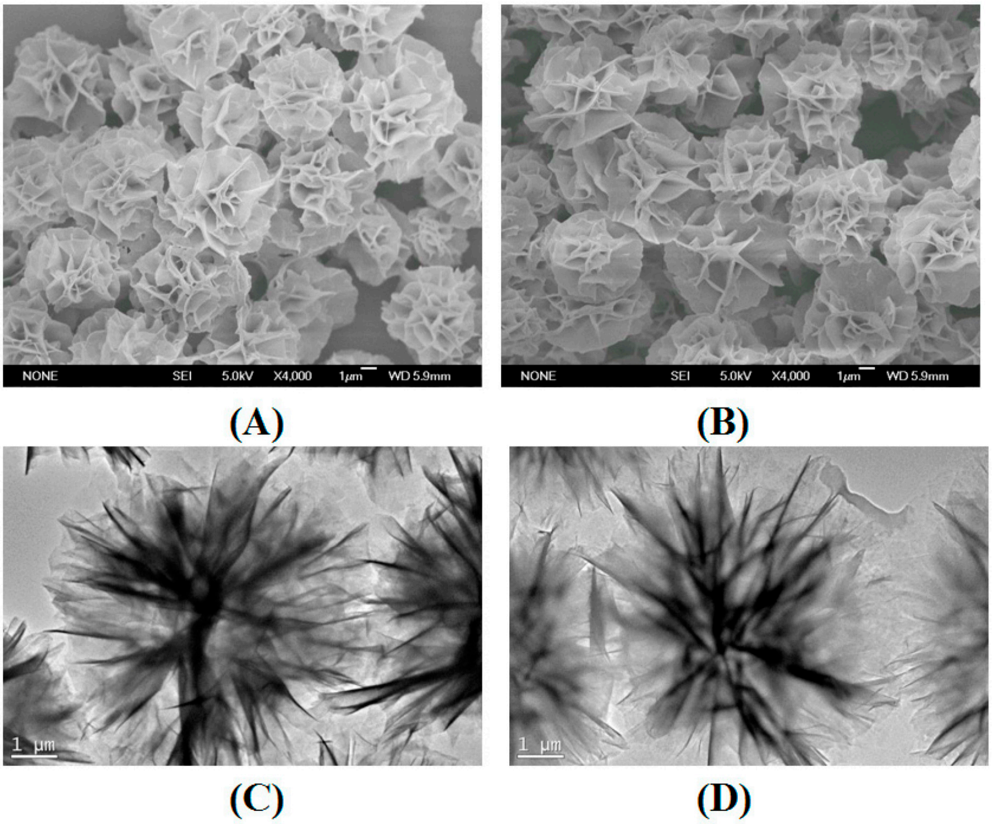

Figure S2. SEM and TEM of the prepared nanobioreactor. (**A**,**C**) The nanobioreactor before the reaction; (**B**,**D**) The recycled nanobioreactor after the tenth reaction batch.
